# An Investigation of the Organoborane/Lewis Base Pairs on the Copolymerization of Propylene Oxide with Succinic Anhydride

**DOI:** 10.3390/molecules25020253

**Published:** 2020-01-08

**Authors:** Lan-Fang Hu, Dan-Jing Chen, Jia-Liang Yang, Xing-Hong Zhang

**Affiliations:** MOE Key Laboratory of Macromolecular Synthesis and Functionalization, Department of Polymer Science and Engineering, Zhejiang University, Hangzhou 310027, China; 11529021@zju.edu.cn (L.-F.H.); 3160103167@zju.edu.cn (D.-J.C.); 11529008@zju.edu.cn (J.-L.Y.)

**Keywords:** succinic anhydride, propylene oxide, aliphatic polyester, organoborane, Lewis base

## Abstract

The copolymerization of biorenewable succinic anhydride (SA) with propylene oxide (PO) is a promising way to synthesize biodegradable aliphatic polyesters. However, the catalytic systems for this reaction still deserve to be explored because the catalytic activity of the reported catalysts and the molecular weights of produced polyesters are unsatisfied. Herein, we investigate the copolymerization of SA with PO catalyzed by the organoborane/base pairs. The types of Lewis bases, organoboranes, and their loadings all have a large impact on the activity and selectivity of the copolymerization. High ester content of >99% was achieved when performed the PO/SA copolymerization using triethyl borane (TEB)/phosphazene base P1-*t*-Bu (*t*-BuP_1_) pair with a molar ratio of 1/1 at 30–80 °C. Using TEB/*t*-BuP_1_ pair with the molar ratio of 4/1 at 80 °C, the turnover of frequency (TOF) was up to 128 h^−1^ and clearly higher than the known TOF values (0.5–34 h^−1^) of the PO/SA copolymerization by previously reported catalysts. The number-average molecular weights (*M*_n_s) of the resultant polyesters reached up to 20.4 kg/mol when copolymerization was carried out using TEB/*t*-BuP_1_ (1/1, in molar ratio) at 30 °C.

## 1. Introduction

Plastic litter, usually made by petroleum-based feedstocks, is an ever-increasing global issue and one of the environmental challenges. Conventional petroleum-based plastic waste is recognized to biodegrade very slowly. Scientists found that plastic waste can break down into ever smaller pieces, become tiny enough to waft in the air and flow in the water and accumulate in organisms or human beings [1,2]. Therefore, it is of great importance to prepare biodegradable materials to replace those plastics.

Aliphatic polyesters are a class of technologically important alternatives to conventional petroleum-based materials because of their biodegradable and the use of renewable resources as feedstock materials [3,4,5]. For example, poly(alkylene succinate)s including poly(ethylene succinate) (PES) and poly(butylene succinate) (PBS) are some of the most important biodegradable polyesters and could be produced by polycondensation of biorenewable resources succinic acid with glycol, butanediol [6,7]. However, the polycondensation method is often energy-intensive and requires removal of small molecules under highly reduced pressure.

**Scheme 1:**
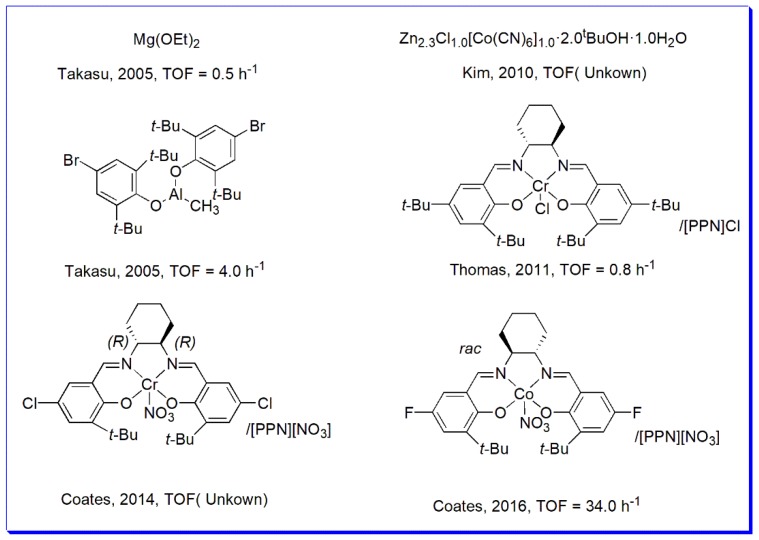
Several catalysts reported for the copolymerization of succinic anhydride (SA) with propylene oxide (PO).

It is of great interest to synthesize polyesters from the copolymerization of epoxides with cyclic anhydrides [8,9,10], because a variety of epoxides and cyclic anhydrides are commercially available and polyesters with various structures can be prepared under mild conditions. Especially, the monomers from renewable resources are intriguing from the environmental viewpoint [11,12,13,14,15,16]. Among various anhydrides studied for the production of polyesters, succinic anhydride (SA) is a kind of promising building block since it is commercially available from renewable resources [6,14,17]. For the copolymerization with epoxide, SA is generally less reactive than other anhydrides, such as maleic anhydride, phthalic anhydride etc., and *M*_n_s of the SA/epoxide copolymers are relatively low (the reported maximum *M*_n_: 9.2 kg/mol [18]) because of the formation of cyclic polymer chains, transesterification of polymer chains or the chain transfer reaction in these systems [19,20]. Therefore, it remains a challenge to develop catalysts with high activity and selectivity for synthesizing high molecular weight SA/epoxide copolymers. Several catalysts such as zinc [19], aluminum [20], chromium [14], cobalt [18,21], and magnesium [20] complexes have been reported to produce alternating propylene oxide (PO)/SA copolymer (Scheme 1). However, the (salen) Co complex showed a maximum turnover of frequency (TOF) of 34 h^−1^ in all the reported catalysts for PO/SA copolymerization [21]. In addition, the metallic residues in polyesters may restrain their application in high-value and sensitive domains, such as biomedical and personal beauty care applications, microelectronic devices, or food packaging.

**Scheme 2:**
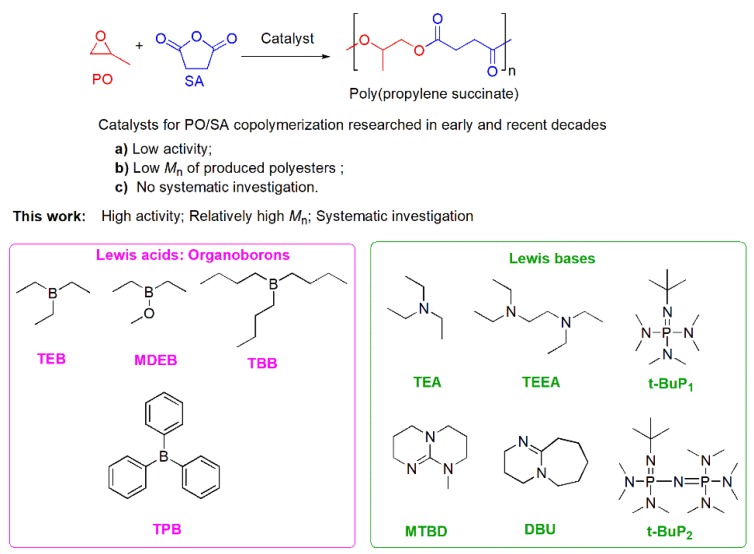
Different catalytic systems for PO/SA copolymerization.

Recently, there has been rapidly growing interest in implementing epoxide-based alternating copolymerization using metal-free catalysts [22,23,24,25,26,27,28,29,30,31,32]. The competence and potential of organocatalysts have been fully verified by the achievements of strictly alternating sequence distribution and high activity. In this work, aiming at efficiently catalyzing the copolymerization of SA with PO that is still rarely investigated, we studied the effect of the structure of organoboranes (OBs) as the Lewis acid (LA), the basicity of Lewis bases (LBs), the LA/LB molar ratios on the activity and selectivity of the copolymerization of SA with PO. We propose a simple organocatalyst system with high catalytic activity (Scheme 2).

## 2. Results

**Figure 1:**
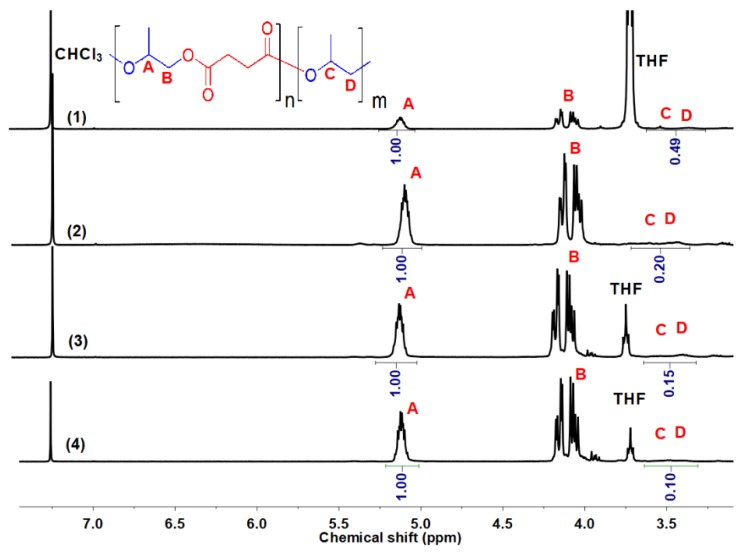
^1^H NMR spectra of crude products at different catalyst systems: (1) trimethylamine (TEA) (entry 1, Table 1); (2) tetraethylethylenediamine (TEEA) (entry 3, Table 1); (3) triethyl borane (TEB)/TEA (entry 2, Table 1); (4) TEB/TEEA (entry 4, Table 1).

**Figure 2:**
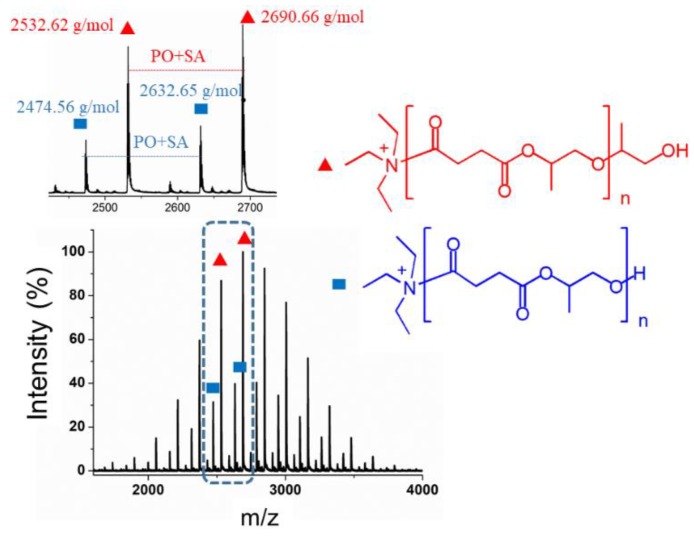
MALDI-TOF-MS spectrum of poly(propylene succinate) produced using TEB/TEA (entry 2, Table 1).

In early years, metal-free initiators, especially the tertiary amines, were used to catalyze the epoxide/anhydride copolymerization [33]. The PO/SA copolymerization was carried out in the presence of triethylamine (TEA) alone (entry 1, Table 1), the result showed a low activity with a TOF of 1 h^−1^ that is similar to the previously reported system [14,20]. Additionally, the ester content of the resultant polyester calculated from the ^1^HNMR spectrum is 86% (Figure 1(1)). We then used triethyl borane (TEB) paired with TEA for the copolymerization (entry 2, Table 1), and the ^1^HNMR spectrum showed that high alternating degree (i.e., ester content: 95%) was achieved (Figure 1(2)). This result is well consistent with the copolymerization of PO/SA by TEB/quaternary onium salt pair (SA conversion: 79%, 45 °C, 10 h) [27]. It was proposed that TEB protected the growing anion to form a tetracoordinate bond-carboxylate (or alkoxide) anion that accelerated the copolymerization [23,27,30]. The matrix-assisted laser desorption/ionization time-of-flight mass (MALDI-TOF-MS) spectrum of the SA/PO copolymer catalyzed by the TEB/TEA pair revealed the -N(CH_2_CH_3_)_3_^+^, -OH terminated copolymer as the major distribution, suggesting that no significant transesterification took place under these conditions (Figure 2). And similar results were also reporeted by Li et al [34]. They observed DMAP^+^, OH-capped polyester chains from MALDI-TOF MS. In addition, inspired by the TEB/double-site base pairs for highly active and living synthesis of carbonyl sulfide (COS)/epoxide copolymers [35], N,N, N′,N′-tetraethylethylenediamine (TEEA) was solely employed to catalyze the PO/SA copolymerization (entry 3, Table 1). The SA conversion was up to 62% within 48 h, and the resultant copolymer had the ester content of 93% that was higher than that of polyester produced by TEA alone (86%, Figure 1(3,4)).

In order to evaluate the relationship of catalytic performance with the structure of the LA/LB pairs, we then studied the effect of the OB/TEEA pairs on the PO/SA copolymerization. The catalysis of the combination of TEEA with OBs (including TPB, TBB, and MDEB, Scheme 2) for the PO/SA copolymerization was investigated, as summarized in Table 1 (entries 4–7). Thereof, TEB/TEEA (1/1) pair led to >99% conversion of SA, and showed higher activity (TOF: 10 h^−1^) and higher selectivity (ester% = 97%) than other OBs companied with 1 equiv TEEA. TOFs of the TEB/TEEA, MDEB/TEEA, TBB/TEEA, TPB/TEEA pairs were 10, 9, 7, and 5 h^−1^, respectively. Correspondingly, the ester contents of the resultant poly(propylene succinate)s were 97%, 94%, 88%, and 85%, respectively. Both activity and selectivity decreased with the order of the steric hindrance of TPB < TBB < MDEB < TEB, while increased with the order of the acidity of TEB > MDEB > TBB > TPB [36,37].

In an attempt to shed light on the effect of LBs on the activity and selectivity for the PO/SA copolymerization, the LBs, *t*-BuP_1_, *t*-BuP_2_, DBU, and MTBD were tested, as summarized in Table 2. The LBs were chosen to span a range of basicity (the pKa values of their conjugate acid LB−H^+^ listed are reported in MeCN, THF, DMSO, or H_2_O, see Table S1 in the Supporting Information)[38,39]: TEEA (pKa^cal^ = 9.03) < TEA (pKa^cal^ = 10.62, pKa^MeCN^ = 18.7) < DBU (pKa^MeCN^ = 24.3) < MTBD (pKa^MeCN^ = 25.4) < *t*-BuP_1_ (pKa^MeCN^ = 26.9) < *t*-BuP_2_ (pKa^MeCN^ = 33.5). TEA, DBU, *t*-BuP_1_ have been previously reported to copolymerize epoxides and anhydrides, but showed low activities [26,28,40]. The equimolar combination of these LBs (TEA, TEEA, *t*-BuP_2_, DBU) with TEB exhibited improved activity towards the PO/SA copolymerization, and the ester contents of the resultant poly(propylene succinate)s were ≥93%. TOFs of these TEB/LB pairs increased with basicity of the bases in the following order: TEA (7 h^−^^1^) < DBU (9 h^−1^) < MTBD (11 h^−1^) < *t*-BuP_1_ (15 h^−1^) < *t*-BuP_2_ (20 h^−1^). However, the situation is different for TEB/TEEA pair, the TOF (10 h^−1^) of TEB/TEEA pair was slightly higher than that of TEB/TEA pair. This is because TEEA is a double-site initiator, which is more efficient to initiate the copolymerization than TEA, although the detail mechanism is still undisclosed [34].

**Figure 3:**
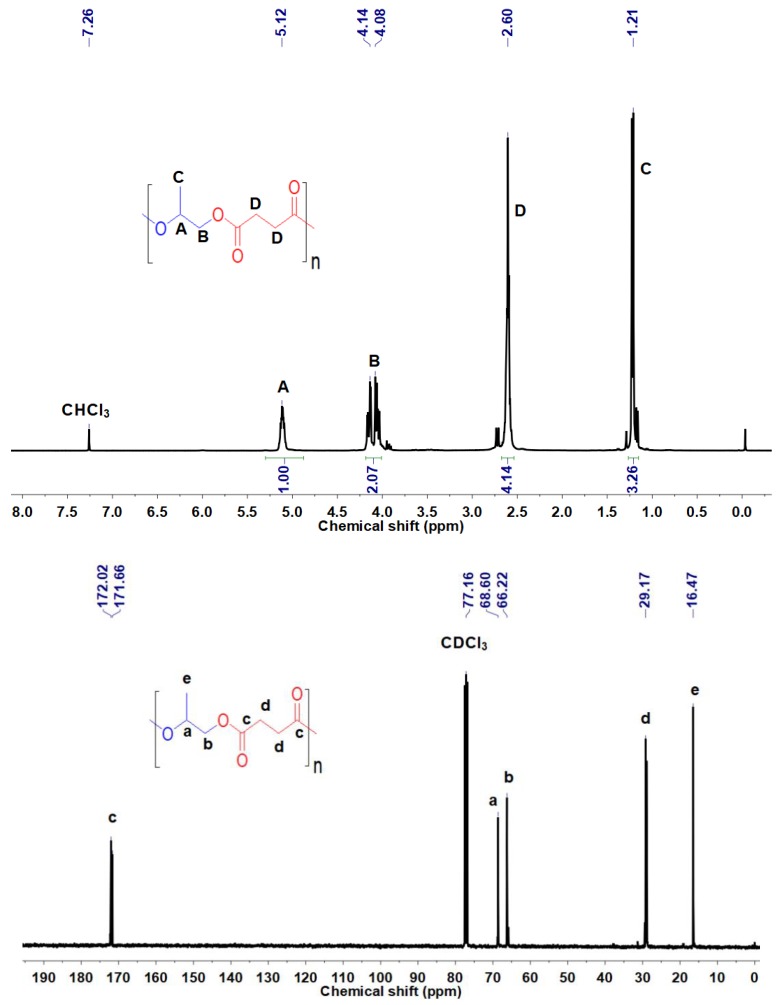
NMR spectra of purified poly(propylene succinate) in CDCl_3_. Top: ^1^H NMR spectrum. Bottom: ^13^C NMR spectrum (entry 1, Table 2).

The TEB/*t*-BuP_2_(*t*-BuP_1_) pairs performed well for the PO/SA copolymerization, and exhibited TOFs of 15–20 h^−1^ (entries 1,2 in Table 2), affording poly(propylene succinate)s with ester content of more than 99%, as shown in Figure 3. The proton resonance of the CH(A) next to the oxygen atom was observed at 5.12 ppm, with the proton signal of the CH_2_(B) groups from PO found in the range 4.08–4.14 ppm. The signal of CH_3_(C) was observed at 1.21 ppm and the signal of CH_2_(D) from SA was observed at 2.60 ppm. No signals of polyether linkages were observed at 3.5–3.8 ppm, which verified the fully alternating structure of poly(propylene succinate). All poly(propylene succinate)s synthesized using various bases in Table 2 have high ester contents of 93% to >99% when the TEB/LB molar ratio was 1/1. It seems that the ester selectivity was less correlated with the basicity of LB. It is reasonable that the production of ether linkage was caused by consecutive attacking to PO which could be mainly inhibited by TEB-masked growing anions [27,32].

The LA/LB feeding ratio greatly affected the activity and selectivity of the PO/SA copolymerization. With the increase of TEB/LB molar ratios, the activity improved but selectivity decreased (entries 2, 4, Table 1, entries 1–4, Table 2 and Table S2). For example, when the molar ratio of the TEB/t-BuP_1_ pair increased from 1/1 to 4/1 at 60 °C, TOF increased from 15 to 59 h^−1^, and the ester contents was 95%. For the TEB/DBU pair-catalyzed PO/SA copolymerization, when the TEB/DBU molar ratio increased from 1/1 to 4/1, TOF values increased from 9 to 33 h^−1^, however, the ester content of the resultant poly(propylene succinate)s sharply decreased from 93% to 37%.

**Figure 4:**
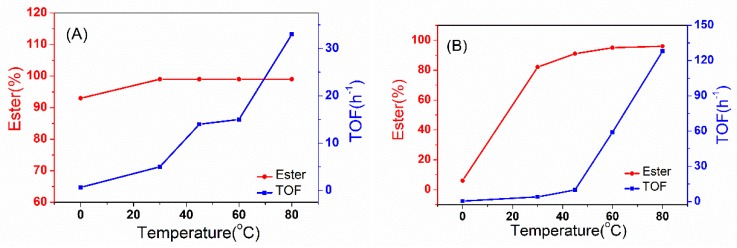
The effect of temperature on turnover of frequency (TOF) values and ester content of the SA/PO copolymerization catalyzed by TEB/*t*-BuP_1_ pairs with the molar ratio of (**A**) 1/1; (**B**) 4/1.

We next investigated the effect of temperature on PO/SA copolymerization catalyzed by the TEB/t-BuP_1_ pair at a wide temperature range from 0 to 80 °C (entries 1, 5, Table 2 and Table S3). As shown in Figure 4, TOFs of the copolymerizations increased significantly with the temperature, and the turning point was about 30–40 °C. Notably, the TOF value was up to 128 h^−1^ at 80 °C in the presence of TEB/t-BuP_1_ pair with the molar ratio of 4/1 (entry 2, Table 2) [21], which is higher than all previously reported TOFs (0.5–34 h^−1^) for the PO/SA copolymerization (Scheme 1) [27]. Moreover, we observed that increasing the reaction temperatures from 30 to 80 °C, the ester contents of all the resultant copolymers were more than 99% when the molar ratio of the TEB/*t*-BuP_1_ pair was 1/1 (Figure 4A) and increased from 76% to 96% when the molar ratio of the TEB/*t*-BuP_1_ pair was 4/1 (Figure 4B). It is reasonable that excess TEB could activate greater amounts of PO, and the TEB-activated PO was easily attacked by the growing species, leading to the formation of ether linkage (Figure 4B) [27,32]. In addition, *M*_n_ of the poly(propylene succinate) decreased with reaction temperatures increased, because of obvious chain transfer reaction at high reaction temperatures. We therefore performed the PO/SA copolymerization with the TEB/*t*-BuP_1_ molar ratio of 1/1 at 30 °C with prolonged time (84 h, entry 2, Table 2), the conversion of SA was up to 98%, and *M*_n_ of the resultant poly(propylene succinate) was high (20.4 kg/mol) (Figure S2), which is close to the calculated value (31.0 kg/mol).

**Scheme 3:**
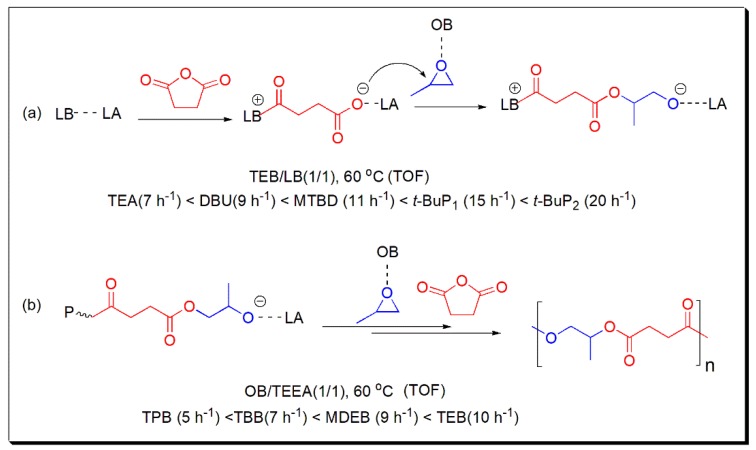
Proposed copolymerization mechanism using the LA/LB pair: (**a**) initiation; (**b**) propagation.

The catalytic mechanism of the LA/LB pair for the copolymerization of epoxide and anhydride has been investigated [26,27,41,42]. For the PO/SA copolymerization, the LB/OB pair firstly reacts with SA, which was determined by MALDI-TOF-MS spectrum (Figure 2). Then the adduct of SA with LA/LB pair generates the zwitterion, which attacks PO for forming growing species (Scheme 3a). For a given OB, the stronger LB was, the faster the initiation step was reached, which results in a higher activity of the PO/SA copolymerization. As our previous reports [27,32], OB could coordinate with the growing carboxylate (or alkoxide) anions when LA/LB molar ratio was 1/1. When excess OB used, OB not only coordinates with the growing carboxylate (or alkoxide) anions for stabilizing them, but also activates PO (Scheme 3b). Therefore, excess OB addition often leads to the contaminant ether linkage and high activity. For a given LB, OB with high acidity and less steric hindrance leads to the higher activity.

## 3. Materials and Methods

### 3.1. Materials

Unless otherwise specified, synthesis operation was carried out on a double-manifold Schlenk vacuum line under nitrogen atmosphere or in a nitrogen-filled glove box. Following purification, materials were stored in a nitrogen-filled glovebox prior to use unless otherwise specified. Triethyl borane (TEB) in THF (1.0 M), triphenylboron (TPB, 0.25 M in THF), Tributylborane (TBB, 1.0 M in THF), diethylmethoxyborane (DEMB) were purchased from Sigma-Aldrich (Shanghai, China) and used without further purifications. 1,8-diazabicyclo [5.4.0] undec-7-ene (DBU) was purified by distillation after stirring with calcium hydride for 3 days. 7-Methyl-1,5,7-triazabicyclo[4.4.0]dec-5-ene (MTBD), phosphazene base P1-*t*-Bu (*t*-BuP_1_; Sigma-Aldrich, 97%), Phosphazene base P2-*t*-Bu (*t*-BuP_2_, 2.0 M in THF) were used as received. Triethylamine (TEA) and tetraethylethylenediamine (TEEA) were purchased from Sigma-Aldrich and purified by distillation after stirring with calcium hydride for 3 days. Propylene oxide (PO) was purified by distillation after stirring with calcium hydride for 3 days. Succinic anhydride (SA) was purchased from Aladdin (Shanghai, China) and purified by sublimation.

### 3.2. Copolymerization of SA with PO, Experimental Details

A 10-mL vial with magnetic stirrer was dried in an oven at 110 °C overnight, then immediately placed into the glovebox chamber. After keeping under vacuum for 2–3 h, the reaction vessel was moved into the glovebox with nitrogen atmosphere. The copolymerization of SA with PO described below is taken from entry 1 in Table 1 as the example. SA (100 mg, 1 mmol) was firstly added into the reactor. Then, PO (0.28 mL, 4 mmol) was added into the vessel after the introduction of the set amount of TEB (10 μL, 0.01 mmol) and TEA (1.9 μL, 0.01 mmol). The vial was sealed with a Teflon lined cap, taken out from the glovebox, and placed in an oil bath. The copolymerization was carried out at 60 °C for 10 h. The reactor was then cooled in ice-water bath, and an aliquot was taken from the crude product for the determination of the ratio of ester/ether linkages by ^1^H NMR spectrum. The collected product was dissolved with dichloromethane and then precipitated in alcohol. The product was collected by centrifugation and dried in vacuum at 40 °C overnight.

### 3.3. Characterization

All ^1^H and ^13^C NMR spectra were recorded on a Bruker AVANCE DMX 400 MHz instrument (Bruker, MA, USA) in CDCl_3_. Chemical shift values were referenced to CHCl_3_ at 7.26 ppm for ^1^H NMR and 77.16 ppm for ^13^C NMR. Molecular weights and molecular weight distributions of the resultant copolymers were determined with a PL-GPC220 chromatograph (Polymer Laboratories, CA, USA) equipped with an HP 1100 pump (Agilent Technologies, CA, USA). The GPC columns were eluted with THF with 1.0 mL/min at 40 °C. The sample concentration was 0.4 wt%, and the injection volume was 50 μL. Calibration was performed using monodisperse polystyrene standards covering the molecular weight range from 500 to 5,000,000 Da.

## 4. Conclusions

In summary, the LA/LB pair is a broad class of active and selective catalysts for the PO/SA copolymerization, which is a biorenewable route to biodegradable polyesters. This work comprehensively disclosed the effect of the structure of the LA/LB pair on the PO/SA copolymerization. The fully alternating copolymers were synthesized in a metal-free catalytic way. The highest activity (TOF: 128 h^−1^) was achieved when the TEB/*t*-BuP_1_ (4/1 in molar ratio) pair was used at 80 °C. *M*_n_ of the resultant poly(propylene succinate) reached up to 20.4 kg/mol using TEB/*t*-BuP_1_ (PO/SA/TEB/*t*-BuP_1_ = 400/200/1/1) at 30 °C. This work demonstrated that the organocatalyst is a powerful tool to catalyze the copolymerization of PO with SA. The development of highly active and selective catalysts for synthesizing a high molecular weight copolymer is ongoing in our lab.

## Figures and Tables

**Table 1:** SA/PO copolymerization catalyzed by LA/LB pairs ^1^.

Entry	LA	LB	Time (h)	SA Conv. (%) ^2^	Ester (%) ^2^	TOF (h^−1^) ^3^	*M*_n_ (kg/mol) ^4^	*Đ* ^4^
1		TEA	48	55	86	1	3.5	1.20
2	TEB	TEA	10	74	95	7	5.1	1.10
3		TEEA	48	62	93	1	3.0	1.22
4	TEB	TEEA	10	>99	97	10	6.4	1.15
5	TBB	TEEA	10	70	88	7	4.1	1.06
6	TPB	TEEA	10	48	85	5	3.3	1.12
7	MDEB	TEEA	10	92	94	9	5.0	1.11

^1^ Reactions were run at 60 °C in neat PO (4 mmol), [PO]/[SA]/[OB]/[LB] = 400/100/1/1. ^2^ SA conversion and Ester were determined by ^1^H NMR spectroscopy of crude reaction mixture. **^3^** TOF = Turnover frequency, mol SA consumed · mol LB^−1^ h^−1^. ^4^ Determined by gel permeation chromatography in THF, calibrated with polystyrene standards.

**Table 2:** SA/PO copolymerization catalyzed by various LBs with TEB ^1^.

Entry	LB	[PO]:[SA]: [TEB]:]LB]	Time (h)	SA Conv. (%) ^2^	Ester (%) ^2^	TOF (h^−1^) ^3^	*M*_n_ (kg/mol) ^4^	*Đ* ^4^
1	*t*-BuP_1_	400:100:1:1	5	73	>99	15	6.0	1.10
2	*t*-BuP_2_	400:100:1:1	5	98	99	20	8.3	1.30
3	DBU	400:100:1:1	5	45	93	9	3.6	1.14
4	MTBD	400:100:1:1	5	57	98	11	4.2	1.13
5 ^5^	*t*-BuP_1_	400:100:4:1	0.5	64	96	128	1.7	1.24
6 ^6^	*t*-BuP_1_	400:200:1:1	84	98	>99	1	20.4	1.42

^1^ Reactions were run at 60 °C in neat PO (4 mmol). ^2^ SA conversion and Ester was determined by ^1^H NMR spectroscopy of crude reaction mixture, **^3^** TOF = Turnover frequency, mol SA consumed·mol LB^−1^·h^−1^. ^4^ Determined by gel permeation chromatography in THF, calibrated with polystyrene standards. ^5^ 80 °C. ^6^ 30 °C.

## Supplementary Materials

The following are available online. Figure S1. Selected GPC curves of poly(propylene succinate)s; Figure S2. GPC curve of the poly(propylene succinate) of enrty 6 in Table 2; Table S1 pKa values of LBs from the Literature; Table S2. SA/PO copolymerization catalyzed by various LBs with TEB; Table S3. SA/PO copolymerization catalyzed by TEB/*t*-BuP_1_ pair at different temperature.
