# Supplementary material for: An Investigation of the Organoborane/Lewis Base Pairs on the Copolymerization of Propylene Oxide with Succinic Anhydride
Source: Molecules. 2020 Jan 8;25(2):253. doi: 10.3390/molecules25020253 (PMC7024261; doi:10.3390/molecules25020253)
Supplement: Supplementary file 1 [file molecules-25-00253-s001.pdf]

*Supporting Information*

# An Investigation of the Organoborane/Lewis Base Pairs on the Copolymerization of Propylene Oxide with Succinic Anhydride

**Lan-Fang Hu, Dan-Jing Chen, Jia-Liang Yang and Xing-Hong Zhang ***

MOE Key Laboratory of Macromolecular Synthesis and Functionalization, Department of Polymer Science and Engineering, Zhejiang University, Hangzhou 310027, China

**Table of Contents**

Table S1. pKa values of LBs from the Literature ........ S2

Table S2. SA/PO copolymerization catalyzed by various LBs with TEB........ S2

Table S3. SA/PO copolymerization catalyzed by TEB/*t*-Bu$P_1$ pair at different temperature........ S2

Figure S1. Selected GPC curves of poly(propylene succinate)s........ S3

Figure S2. GPC curve of the poly(propylene succinate) of enrty 6 in Table 2. ........ S3

**Table 1.** pKa values of LBs from the Literature [1].

| LB | pKa, MeCN | pKa, THF | pKa, DMSO | pKa, $H_2O$ | pKa, cal |
|---|---|---|---|---|---|
| TEA | 18.7 | 12.5 | 9.0 | 10.7 | 10.62 |
| TEEA | | | | | 9.03 |
| MTBD | 25.4 | 17.9 | | 13.0 | 14.37 |
| DBU | 24.3 | 16.8 | 13.9 | 11.9 | 13.28 |
| $t$-Bu$P_1$ | 26.9 | | 15.7 | | |
| $t$-Bu$P_2$ | 33.5 | | 21.5 | | |

pKa, cals were calculated using Advanced Chemistry Development (ACD/Labs) Software V11.02(©1994-2019 ACD/Labs), $pK_a$ values of $t$-Bu$P_1$ and $t$-Bu$P_2$ are collected from "R. Schwesinger, et al., Liebigs Ann. 1996, 1055".

**Table 2.** SA/PO copolymerization catalyzed by various LBs with TEB. [1.]

| Entry | LB | PO:SA:TEB:LB | Time (h) | SA Conv. (%) [2] | Ester (%) [2] | TOF($h^{-1}$) [3] | $M_n$(kg/mol) [4] | $Đ$ [4] |
|---|---|---|---|---|---|---|---|---|
| 1 | TEA | 400:100:4:1 | 3 | 50 | 87 | 17 | 4.1 | 1.18 |
| 2 | TEEA | 400:100:4:1 | 3 | 72 | 92 | 24 | 3.5 | 1.13 |
| 4 | $t$-Bu$P_1$ | 400:100:4:1 | 1.5 | 89 | 95 | 59 | 5.0 | 1.15 |
| 6 | $t$-Bu$P_2$ | 400:100:4:1 | 1.5 | 56 | 81 | 37 | 1.7 | 1.14 |
| 8 | DBU | 400:100:4:1 | 3 | >99 | 37 | 33 | 8.2 | 1.16 |
| 10 | MTBD | 400:100:4:1 | 3 | 67 | 92 | 22 | 4.5 | 1.14 |

[1] Reactions were run at 60 °C in neat PO (4 mmol). [2] SA conversion and Ester was determined by $^1$H NMR spectroscopy of crude reaction mixture. [3] TOF = turnover of frequency, (Mol SA consumed)/(mol LB h). [4] Determined by gel permeation chromatography in THF, calibrated with polystyrene standards.

**Table 3.** SA/PO copolymerization catalyzed by TEB/$t$-Bu$P_1$ pair at different temperature. [1.]

| Entry | [PO]:[SA]: [TE]B: [$t$-Bu$P_1$] | Temp. (°C) | Time (h) | SA Conv. (%) [2] | Ester (%) [2] | TOF ($h^{-1}$) [3] | $M_n$ (kg/mol) [4] | $Đ$ [4] |
|---|---|---|---|---|---|---|---|---|
| 1 | 400:100:4:1 | 60 | 1.5 | 89 | 95 | 59 | 5.0 | 1.15 |
| 2 | 400:100:4:1 | 45 | 5 | 52 | 82 | 10 | 1.1 | 1.35 |
| 3 | 400:100:4:1 | 30 | 24 | 85 | 76 | 4 | 2.3 | 1.33 |
| 4 | 400:100:4:1 | 0 | 24 | 13 | 6 | <1 | - | - |
| 5 | 400:100:1:1 | 80 | 3 | >99 | >99 | 33 | 2.9 | 1.29 |
| 6 | 400:100:1:1 | 45 | 5 | 69 | >99 | 14 | 5.8 | 1.11 |
| 7 | 400:100:1:1 | 30 | 16 | 83 | >99 | 5 | 7.7 | 1.14 |
| 8 | 400:100:1:1 | 0 | 48 | 31 | 93 | 0.6 | 5.2 | 1.11 |

[1] Reactions were run in neat PO. [2] SA conversion and Ester was determined by $^1$H NMR spectroscopy of crude reaction mixture. [3] TOF = turnover of frequency, (Mol SA consumed)/(mol LB h). [4] Determined by gel permeation chromatography in THF, calibrated with polystyrene standards.

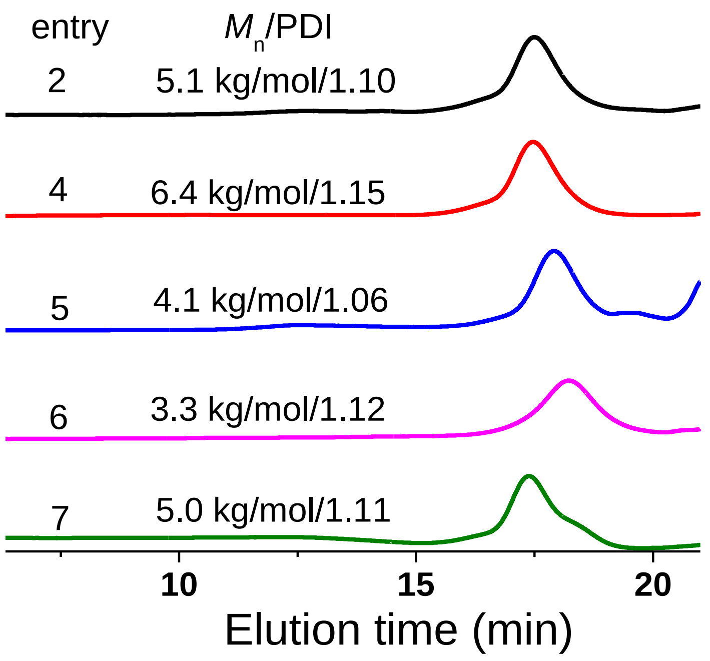


**Figure 1.** Selected GPC curves of poly(propylene succinate)s.(entries 2, 4–7 in Table 1).

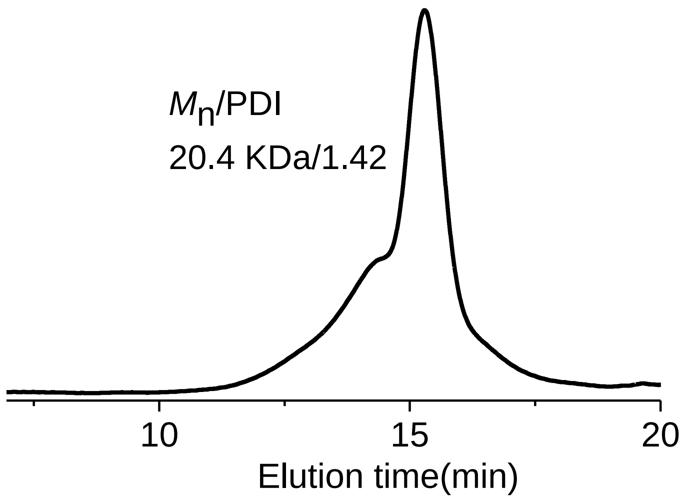


**Figure 2.** GPC curve of the poly(propylene succinate) of enrty 6 in Table 2.

**Reference:**

1. Lin, B.; Waymouth, R.M. Organic Ring-Opening Polymerization Catalysts: Reactivity Control by Balancing Acidity. *Macromolecules* **2018**, *51*, 2932–2938. doi:10.1021/acs.macromol.8b00540.
